# *Vatairea* Genus as a Potential Therapeutic Agent—A Comprehensive Review of Ethnobotanical, Phytochemical, and Pharmacological Properties

**DOI:** 10.3390/ph18030422

**Published:** 2025-03-17

**Authors:** Sarah Andrade Toledo, Laryssa Danielle da Silva Reis, Brenda Costa da Conceição, Lucas Villar Pedrosa da Silva Pantoja, Fábio José Coelho de Souza-Junior, Flávia Cristina Santos Garcez, Cristiane Socorro Ferraz Maia, Eneas Andrade Fontes-Junior

**Affiliations:** Laboratory of Pharmacology of Inflammation and Behavior, Institute of Health Sciences, Federal University of Pará, Belém 66075110, Brazil

**Keywords:** *Vatairea* sp., folk medicine, phytochemical, pharmacology, ethnobotany

## Abstract

The *Vatairea* genus (Fabaceae family) is widespread in the Amazon rainforest. Some species of this genus are known for their ethnobotanical significance and biological potential. The present study explores the pharmacological and promising therapeutic activities, ethnobotanical profile, and phytochemical prospection of *Vatairea* sp., a monophyletic group of flowering plants, which includes economically and culturally important genera due to their diverse uses, including medicinal applications. *V. lundellii*, *V. guianensis*, *V. erythrocarpa*, *V. fusca*, *V. heteroptera*, *V. paraensis*, *V. sericea*, and *V. macrocarpa* are included in the *Vatairea* sp., also recognized for its high wood quality and potential medicinal properties. Studies show significant antibacterial activity in *V. guianensis* extracts against Gram-positive and Gram-negative bacteria, whereas *V. macrocarpa* lectin exhibits broad-spectrum antibacterial effects, including modulation of antibiotic resistance. Additionally, *V. macrocarpa* and *V. guianensis* have demonstrated antifungal properties, with compounds like Vatacarpan exhibiting potent activity against *Candida* sp. In vivo studies highlight the neurotoxic effects of *V. macrocarpa* lectin, suggesting a dual role in the central nervous system. Despite these findings, research on Vatairea’s toxicological aspects is limited, with only a few studies on *V. macrocarpa* and *V. guianensis* extracts indicating a need for further exploration of this genus’ pharmacological and therapeutic potential.

## 1. Introduction

Traditional medicine has supplied a foundation for pharmacology for decades, contributing to drug discovery and development. The Amazon rainforest, rich in biodiversity and folk medicine, is a crucial resource for discovering bioactive compounds [1].

In Amazon plants, the *Vatairea* genus has received significant prominence. The genus is a non-endemic common plant in Brazil, composed of eight species and classified in the family Leguminosae (Fabaceae). *Vatairea* sp. is also present in other Amazon countries, such as Peru, Venezuela, and Colombia. Plants of *Vatairea* sp. are popularly known as amargoso (bitter), angelim-amargoso (bitter angelim), angelim-do-igapó, guáboa, angelim [2]. In traditional communities, these botanical species’ heartwood and bark are prepared by maceration and utilized for gastrointestinal diseases, insulin control, and other functions; however, solely two of the eight species have been studied for pharmacological activities (i.e., V. *guianensis* and *V. macrocarpa*). Their ethnopharmacological therapeutic uses in Brazil involve treating digestive and circulatory systems diseases [3,4].

This comprehensive review aims to systematize the existing knowledge and provide an in-depth and critical analysis of the genus *Vatairea*, highlighting its pharmacological and therapeutic potential, ethnobotanical significance, and phytochemical properties. Our pioneering approach also highlights existing gaps, providing insights into future research. For this purpose, studies on PubMed, Scopus, and Web of Science platforms were selected without language restrictions. The scientific names of the *Vatairea* species and their scientific synonyms (World Flora Online database) were used as search keys. Articles related to the plant’s therapeutic, pharmacological, ethnobiological, and phytochemical aspects were selected.

## 2. Ethnobotanical Features

### 2.1. Taxonomy and Botanical Aspects

Recognized as the most diversified family in terms of Brazilian flora and one of the largest angiosperm families, the Fabaceae family comprises 19,325 species divided into 727 genera worldwide, with 2807 species across 222 genera found in Brazil [5]. The Fabaceae family is also a monophyletic group of flowering plants, popularly known as leguminous, ranging from trees to shrubs. It plays a pivotal role in nitrogen fixation through its relationship with the soil. The family is divided into three subfamilies: Caesalpinioideae, Mimosoideae, and Faboideae [6]. Some genera within the Fabaceae family stand out for economic and cultural reasons due to their high diversity and versatile uses, ranging from food and construction to medicinal purposes [7].

**Figure 1:**
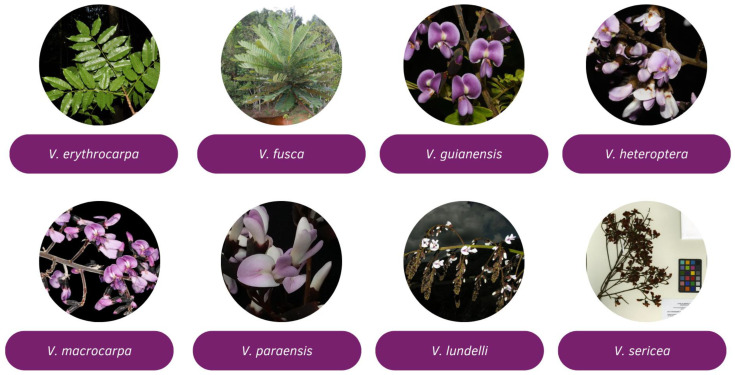
Photographs of the *Vatairea* species. The images of *V. erythrocarpa*, *V. fusca*, *V. guianensis*, *V. heteroptera*, and *V. macrocarpa* were provided by Domingos Cardoso and are sourced from the Reflora website [2]. The photograph of *V. paraensis* is credited to L. O. A. Teixeira, also from the *Reflora* website [2]. Images of *V. lundelli* and *V. sericea* were obtained from the *Trópicos* website, with photos by Aguilar Fernandez [10] and MBG [11].

*Vatairea* is one of the primary genera in the family, known for its neotropical origin and excellent wood quality, comprising eight species: *Vatairea lundellii*, *V. erythrocarpa*, *V. fusca*, *V. guianensis*, *V. heteroptera*, *V. macrocarpa*, *V. paraensis*, and *V. sericea* [8] (Figure 1). These species share some similarities, such as spirally grouped leaves at the top of their branches and petals ranging from shades of blue to purple, with their primary life form being trees [9]. The genus has garnered scientific interest due to its diverse applications, including its potential medicinal uses, reflecting the significant subject for this comprehensive review.

*V. heteroptera* is endemic to Brazil and unique in distribution in the Atlantic Forest. It is also a predominantly large tree, averaging 20–30 m in height, and heartwood tends towards brown, with a trunk that has barely perceptible sapwood at the beginning. *V. erythrocarpa* grows more towards the central side of the Amazon. It presents a red epithet commonly over 20–30 m tall, with a more yellowish-brown interior [8].

*V. fusca* also varies between 20 and 30 m in height, with its brown heartwood, seed, and embryo difficult to identify. Such species are abundant in the lower Amazon region and southern Pará state. *V. lundellii* is over 20 m tall, with yellowish-brown heartwood, and blooms relatively during drier periods, such as January to March, with its fruit growing soon afterward. Its geographical occupation is also characteristic of southern Mexico, predominantly in rainforests [8].

*V. guianensis* is recurrent throughout the Amazon River region, accompanied by floodplain forests; it is of medium size, 8–25 m; has easy recognition through the size of its flowers, which reach up to 3.5 cm; and huge sapopemas at the beginning of the trunk, being considered the most geographically distributed. *V. macrocarpa* is 5–12 m high, geographically native with greater predominance in the Brazilian Cerrado region. This plant presents a slightly thinner bark, associated with a brown heartwood, and enormous phenotypic plasticity, with specific variations related to the soil [8].

*V. paraensis*, the largest tree (30–40 m in height) in the *Vatairea* genus, presents huge sapopemas on the trunk base and rougher bark, resembling *V. fusca*. Other trees differ from this, however, due to its more turbocharged hypanthus [8]. Finally, predominantly located in the rainforest, *V. sericea* is also a large tree (20–40 m), with uncommonly sharper leaflets, small sapopemas on the trunk, a rougher surface, and more yellowish heartwood [8].

### 2.2. Distribution and Traditional Uses

*Vatairea* sp. are native to Brazil and endemic in Guyana and the Atlantic coast of Central America and Mexico [8]. In Brazil, the species are found in the Amazon (*V. erythroderma*, *V. fusca*, *V. guianensis*, *V. macrocarpa*, *V. paraensis*, and *V. sericea*), Cerrado, Caatinga (*V. macrocarpa*), and in the Atlantic Forest (*V. heteroptera* and *V. lundellii*) [8,12].

**Figure 2:**
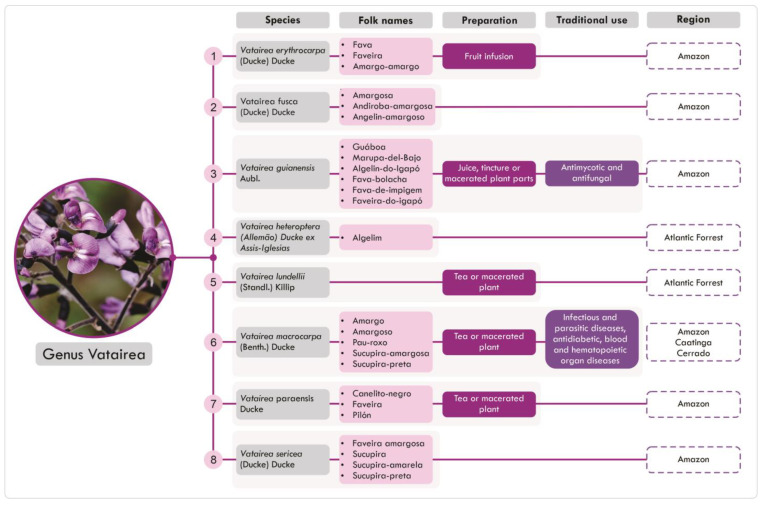
Popular names, preparation, traditional uses, and geographical distribution of *Vatairea* sp.

Plants of the genus are commonly known in Brazil as ‘faveira’, ‘fava’, ‘fava-amarela’, ‘sucupira’, ‘sucupira-amarela’, ‘sucupira-preta’, ‘sucupira-amargosa’, ‘bittersa’, ‘andiroba-amargos’, ‘angelim-de-igapó’, ‘angelim’, ‘angelim-amargoso’, ‘angelim-do-cerrado’, ‘pau-roxo’ (Figure 2) [2,13].

According to ethnopharmacological information, plants of the *Vatairea* genus are universally used for dermatological treatments. *V. guianensis* is used in the form of juice, tincture, or maceration of the fruit, bark, stem, or root, to treat superficial mycoses and skin diseases [13,14,15,16]. It also corresponds to the most cited species in the reviewed literature, accompanied by the species *V. macrocarpa* and *V. lundellii*, which also have antimicrobial potential [16,17,18,19,20,21,22].

Anti-inflammatory and antiproliferative activities have also been associated with the *V. guianensis* and *V. macrocarpa* species [22,23,24,25]. *V. macrocarpa* also helps to control diabetes and treat stomach problems [23,26,27,28].

Thus, the eight species of *Vatairea*, native to Brazil, often have similar popular names and applications in traditional medicine, related to treating infections and inflammatory processes, but also applied to treating endocrine diseases. The preparation forms observed are also diverse, including juices, tinctures, teas, and macerations, which may indicate different solubility patterns to the diverse medicinal properties reported by traditional people [2,4,13,22,23,24].

## 3. Phytochemical Aspects

Only three of eight *Vatairea* species were subjected to phytochemical studies, namely *V. guianensis*, *V. macrocarpa*, and *V. heteroptera*, whose findings are summarized in Table 1 and are discussed below.

### 3.1. Vatairea guianensis

Simatupang et al. [29] conducted an illuminating phytochemical investigation of *V. guianensis* to identify potential chemical compounds related to the skin-irritating effects of the wood derived from this species. It is worth highlighting that species of the genus *Vatairea* have significant economic value due to the commercialization of trunk wood, which was extensively exported to Europe in past decades. Simatupang and colleagues [29] identified three anthrone compounds in the benzene extract of the heartwood of *V. guianensis* for the first time, namely chrysophanic acid-9-anthrone, physcion-9-anthrone, and physcion-10-anthrone (Table 1). Some anthrone derivatives have been shown to cause skin irritation [32].

Following Simatupang et al. [29]’s study, Piedade and Wolter Filho [14] identified a new anthraquinone from *V. guianensis*, called physcion. The authors employed a different phytochemical approach, using a hot extraction with a Soxhlet apparatus to obtain an ethanolic extract, fractionated with organic solvents (e.g., hexane, benzene, and methanol). The benzene fraction allowed the isolation of physcion, chrysophanol, emodin, and dihydromacarfinic acid (a triterpene compound). This successful phytochemical approach led to the isolation of significant quantities of these compounds (Table 1).

The first phytochemical study with the species *V. guianensis* was published only in 2011, based on the essential oil of its fruits. Chemical analysis identified the prevalence of aldehydes and carboxylic acids, including docosahexaenoic acid (DHA), an omega-3 fatty acid known for its role in neurodevelopment [18,33]. On the other hand, analysis of the ethanolic extract demonstrated the presence of anthraquinone compounds, chrysophanol, and physcion, confirming the previously identified species [18].

In addition, four new isoflavonoid compounds from *V. guianensis* leaves were identified: 5,3′-dihydroxy-4′-methoxy-2″,2″-dimethylpyrano-(5″,6″:8,7)-isoflavone, 5,7-dihydroxy-3′,4′-methylenedioxy-8-prenyl-isoflavone, 5,3′-dihydroxy-4′-methoxy-7-O-β-glucopyranoside-8-prenyl-isoflavone, and derrone [19]. The authors suggested that this phytochemical profile was closely related to the significant antioxidant properties exhibited by this plant. In this context, Souza and coworkers [30] also assessed the chemical profile of the ethanolic extract of *V. guianensis*, investigating the sapwood part. Fractionation of the ethanolic extract with hexane yielded the isolation of chrysophanol, physcion, formononetin, bolusantol D, betulinic acid, sitosterol, and stigmasterol. It is important to highlight that formononetin and bolusantol D had been previously reported for species of the Fabaceae family, but this was the first report of their occurrence in the *Vatairea* genus.

The most recent scientific report on the phytochemical profile of *V. guianensis* was also conducted by Souza and colleagues [22]. In a scenario focused on flavonoid identification, they revealed a novel isoflavonoid obtained from the ethanolic extract of *V. guianensis* leaves, which was fractionated with ethyl acetate. The identified isoflavonoid compounds were 5,7,3′-trihydroxy-4′-methoxy-8-prenyl-isoflavone (a new compound identified in the species), lupiwighteone, and 5,7,4′-trihydroxy-3′-methoxy-8-prenylisoflavone.

Albeit lectins are not part of the secondary metabolism in plants, their pivotal role in biological systems has increased scientific interest, particularly in species of the genus *Vatairea*. Remarkably, these proteins can recognize and bind to specific carbohydrates, mediating essential immunological processes such as pathogen defense and cell signaling. Lectins are present in plants and animals and can have various applications in medical research, such as in diagnosis, antiviral, antitumor drugs, and drug delivery systems [34,35,36,37]. Thus, new lectins with benefits in these areas have been extensively investigated. Lectins from the *Vatairea* genus, specifically *V. guianensis*, have been successfully isolated and studied, revealing key structural and functional properties. The *V. guianensis* lectin exhibits high thermal stability and remains stable across all-around acidic and basic pH levels. Its glycoprotein structure resembles known lectins, including *V. macrocarpa* [38,39].

### 3.2. Vatairea macrocarpa

The limited phytochemical investigations of *V. macrocarpa* reveal a predominant presence of phenolic compounds, particularly catechin, epicatechin, kaempferol-3-O-α-l-rhamnopyranoside, tannins, and other phenolic substances [23]. Santana et al. [19] conducted a comprehensive phytochemical analysis, preparing eight extracts from various parts of the plant using hexane, ethyl acetate, and ethanol as solvents. The extracts were obtained from root wood (hexane and ethyl acetate), root bark (ethyl acetate), stem wood (hexane, ethyl acetate, and ethanol), stem bark (ethyl acetate), and leaves (ethyl acetate). From the ethyl acetate extract of the root bark, several compounds were isolated following fractionation with hexane, ethyl acetate, and methanol. Notably, a new pterocarpan was identified and named Vatacarpan by the authors, along with musizin, a naphthoquinone previously reported in the literature.

The lectin from *V. macrocarpa* has also been a focus of the investigation. This lectin was identified with a 22 kDa N-terminal sequence, a structural feature comparable to other endogenous protein fragments, suggesting potential shared functional roles [38,39].

### 3.3. Vatairea heteroptera

Formiga et al. [30] investigated the phytochemical composition of *V. heteroptera*, focusing on the trunk wood, which was extracted using benzene. The study identified several key compounds: chrysophanol, sitosterol, stigmasterol, emodin, (2S)-7-hydroxyflavone, and formononetin. This report is notable for being the first to document phytochemicals with potential medicinal value in the genus *Vatairea*. Specifically, the study identified anthraquinones (chrysophanol and emodin), flavonoids ((2S)-7-hydroxyflavone and formononetin), and triterpenoids (sitosterol and stigmasterol) in the genus.

Thus, there is a marked lack of information on the phytochemical composition of *Vatairea* sp. In the few studies carried out, aldehydes and carboxylic acids (essential oil) were identified: lectins (seeds) [18,40,41,42], which will be discussed in more detail later, given the interest in the literature on these substances; anthraquinones, flavonoids, triterpenes, and tannins (extracts from various parts of plants), related to the antioxidant potential of some of these species [14,19,22,24,30]. These elements need to be further investigated, and new studies need to be conducted to increase knowledge of these species’ potential and the safety of their use.

## 4. Pharmacological Properties

There are limited studies on the safety of using *Vatairea* species for therapeutic purposes. Despite this, its effects on microorganisms and diseases related to the central nervous, endocrine, immune, cardiovascular, and renal systems have already been explored. The investigation, however, is still in its initial stages. Studies related to the biological properties of *Vatairea* species are summarized in Table 2 and will be discussed below.

### 4.1. Toxicity Studies

As mentioned above, only two species (*V. macrocarpa* and *V. guianensis*) have been evaluated for their toxicity. *V. macrocarpa* leaves’ ethanolic extract did not show cytotoxicity in neutrophils culture [24]. No adverse effects in rodents, such as relevant behavioral changes or deaths, were observed at subchronic doses [44]. On the other hand, the lectin from *V. macrocarpa* showed in vitro cytotoxicity in human lymphocytes at concentrations above 0.5 µM and demonstrated genotoxicity at concentrations above 8.0 µM [43].

For *V. guianensis*, the hydroethanolic extract obtained by maceration showed no acute oral toxicity at doses below 2000 mg/kg [16]. Thus, the studies indicate that extracts of *V. macrocarpa* and *V. guianensis* are relatively safe, noting that they only cover in vitro tests and murine models. It is also important to highlight the cytotoxic and genotoxic effects of the *V. macrocarpa* lectin. Therefore, there is a need for more in-depth investigations to validate the safety of its use, since they are widely used in traditional medicine. It is also important to highlight the need to investigate the toxicity of the other six species of the genus, for which no studies were identified.

### 4.2. Pharmacological Studies

Although the *Vatairea* genus comprises diverse species, most studies investigating pharmacological activity primarily focused on *V. macrocarpa* and *V. guianensis*. Numerous models have been used to investigate their pharmacological activities and therapeutic potential. In vitro studies gathered findings that underscore the importance of studying medicinal plants, such as, in this case, species of the *Vatairea* genus.

It is noted that predominantly in vivo studies have demonstrated the pharmacological activities derived from various species of the genus *Vatairea*. Molecules such as lectins, found in several species including *V. macrocarpa* and *V. guianensis*, present numerous opportunities for modulating biological systems [52].

#### 4.2.1. Antibacterial Activity

Antibacterial activity of several *V. guianensis* extracts (e.g., hydroalcoholic, hexane, chloroform, and methanolic) against Gram-positive (*Staphylococcus aureus* and *Enterococcus faecalis*) and Gram-negative (*Pseudomonas aeruginosa* and *Salmonella* sp.) bacteria have been reported [45]. All extracts demonstrated such activity, with minimum inhibitory concentration (MIC) ranging from 3.12 to 50 µg/mL and minimum bactericidal concentration (MBC) ranging from 6.25 to 100 µg/mL. Additionally, Oliveira et al. [17] confirmed the effectiveness of *V. guianensis* against *S. aureus*, further supporting its antibacterial properties.

Anthraquinones are well-documented for their significant antimicrobial potential [53]. Within the *Vatairea* genus, particularly in *V. guianensis*, notable anthraquinone compounds such as chrysophanol, physcion, and their derivatives have been identified, likely contributing to the antimicrobial activity reported [22,30,31]. Physcion, a natural anthraquinone derivative, exhibits remarkable antibacterial activity against *S. aureus*, *S. epidermidis*, and *P. aeruginosa*. At the same time, chrysophanol has also been consistently highlighted for its potent antimicrobial properties against various bacterial and fungal strains [54,55,56]. These findings revealed how phytochemical investigations are essential for advancing pharmacological research on natural products, as they enable the identification of bioactive compounds and provide critical insights into their therapeutic potential, paving the way for the discovery of novel antimicrobial agents.

Unlike *V. guianensis*, for which research focuses on antibacterial activity in extracts, *V. macrocarpa* has been extensively studied for its lectin, a protein extracted from the seed. Vasconcelos et al. [40] demonstrate the broad-spectrum antibacterial activity of *V. macrocarpa* lectin against several pathogens, including S*. epidermidis*, *S. aureus*, *Klebsiella oxytoca*, and *P. aeruginosa*. Similarly, Teixeira [41] found that lectins from different plant species, including *V. macrocarpa*, reduced the adhesion of *Streptococcus* sp. to the salivary components of dental plaque. These findings suggest that the *V. macrocarpa* lectin, like the lectins from other species, can disrupt bacterial adhesion, potentially preventing biofilm formation. This potential antibacterial activity of *V. macrocarpa* seed lectins was tested against multi-drug-resistant bacteria strains of *S. aureus* and *E. coli* [42]. The *V. macrocarpa* lectin demonstrated the ability to modulate antibiotic activity [57]. When combined with antibiotics, such as gentamicin, norfloxacin, and penicillin, the lectin increased the effectiveness against *S. aureus*, but not against *E. coli* [42]. In silico predictions suggest that this interaction between the lectin and gentamicin involves residues within the lectin’s carbohydrate-binding site [58]. This finding reinforces the potential of *V. macrocarpa* lectins as a promising strategy for combating multi-resistant bacteria.

#### 4.2.2. Antifungal Activity

In addition to antibacterial properties, *V. guianensis* and *V. macrocarpa* have also been explored for their antifungal potential. Santana et al. [20] investigated in vitro the antifungal activity of several Fabaceae species, including *V. macrocarpa*. In this study, the authors explore the ethyl acetate extract of *V. macrocarpa* and evaluate fractions and isolated compounds. Notably, the isolated compound vatacarpan demonstrated significant in vitro activity against *Candida albicans* (MIC = 0.98 µg/mL), a remarkably low concentration compared to established antifungal drugs, such as fluconazole and amphotericin B. This finding highlights the potential of *V. macrocarpa* as a source for developing new antifungal agents. Recently, in a study on antifungal activity focusing on *V. guianensis*, Souza et al. [22] isolated a new compound, 5,7,3′-trihydroxy-4′-methoxy-8-prenylisoflavone, from leaves, and evaluated its antifungal properties. The authors employed the ethanolic extract of *V. guianensis* leaves against several *Candida* species, including *C. dubliniensis*, *C. albicans*, *C. parapsilosis*, and *C. krusei*. The results demonstrated the antifungal activity of the extract against *C. dubliniensis*, *C. albicans*, and *C. krusei*. Remarkably, the extract fraction exhibited significant activity against all fungi investigated, while the isolated compound showed more potent activity against *C. parapsilosis* and *C. dubliniensis*.

The evidence gathered demonstrates the potential of *V. macrocarpa* and *V. guianensis* for treating infectious conditions, presenting a broad spectrum of action, combating bacteria and fungi, and enhancing the activity of conventional antibiotics. These findings are interestingly aligned with the traditional use of these plants to treat skin diseases, conditions that can be explored in in vitro and murine models, to expand the pharmacological characterization of the species and elucidate its potential benefits [52]. No studies were identified regarding the possible antibiotic properties of the other *Vatairea* species, highlighting yet another gap that must be filled.

#### 4.2.3. Endocrine System

Among the species of the *Vatairea* genus, *V. macrocarpa* stands out for its endocrine-modulating effects. In streptozotocin-challenged rats, ethanolic extracts of *V. macrocarpa* demonstrated significant anti-diabetic activity by improving insulin resistance, with authors suggesting a possible modulation of pancreatic beta-cell function [26]. A recent study employing refined pharmacological techniques found that *V. macrocarpa* stem bark ethanolic extract attenuated diabetes-like parameters by reducing glycemia, urinary glucose, and urea levels. The extract improved insulin signaling by enhancing GLUT4 receptor translocation to the cell membrane and decreasing phosphoenolpyruvate carboxykinase (PEPCK) activity, thereby reducing gluconeogenesis [27]. These findings, combined with the phenolic-rich composition of *V. macrocarpa*, highlight its potential as a source of endocrine-active compounds.

#### 4.2.4. Cardiovascular and Renal Systems

*V. macrocarpa* galactose-binding lectin from seeds significantly increases perfusion pressure, renal vascular resistance, urinary flow, and glomerular filtration rate, not altering the tubular transport of sodium, potassium, or chloride in isolated Wistar rats’ kidneys [46]. Pretreatment with a lectin–galactose complex blocked these renal parameters’ increase, suggesting that the lectin specifically interacts with carbohydrate-binding sites in the renal system. Additionally, kidneys treated with the lectin showed moderate deposits of proteinaceous material in the tubules and urinary space, while those pretreated with the lectin–galactose complex showed only small amounts of this material, with no abnormalities in the renal tubules. These results indicate that *V. macrocarpa* lectin significantly affects renal function, modulated by its interaction with carbohydrates [46].

*V. guianensis* lectin exhibits vasorelaxant ex vivo activity in contracted rat aortas. This effect is strictly endothelium-dependent and involves nitric oxide production and the lectin domain [38]. Interestingly, despite the structural similarity, *V. macrocarpa* and *V. guianensis* lectins demonstrated divergent effects on the vascular bed. *V. macrocarpa* lectin demonstrated vasoconstrictive properties on the renal artery, whereas *V. guianensis* lectin exhibited a vasorelaxant effect on the aorta. This peculiarity should be further explored to elucidate the structure–activity relationship and the mechanisms that trigger such effects [38,46].

#### 4.2.5. Immune System

Anti-inflammatory properties of the ethanolic leaf extract of *V. macrocarpa* in experimental in vitro models were also described. The extract exhibits phenolic compounds and flavonoids, such as catechin, epicatechin, and kaempferol-3-O-a-l-rhamnopyranoside. The in vivo model was effective in reducing leukocyte migration and protein exudation in carrageenan-induced pleurisy, as well as inhibiting inflammatory parameters in models of persistent paw inflammation and bacillus Calmette–Guérin (BCG)-induced pleurisy. These findings suggest that *V. macrocarpa* extract has potential as a natural agent for treating inflammatory diseases [24].

Interestingly, Alencar et al. [47] showed that *V. macrocarpa* seed lectins can induce an inflammatory response. The lectin was injected into the peritoneum of rats to investigate how it triggers this effect, resulting in increased neutrophil migration into the peritoneal cavity. However, adding α-D-galactose to the lectin solution partially inhibited this effect. Neutrophil migration was also reduced when the peritoneal cavity was depleted from resident cells previously, suggesting an indirect mechanism of neutrophil migration, possibly mediated by resident macrophages. The authors propose that galactose-binding lectins’ effects are not linked to inflammatory mediators like lipoxygenase, cyclooxygenase, or platelet-activating factor (PAF). On the other hand, they observed that dexamethasone and thalidomide reduce lectin-induced neutrophil migration, indicating the possible involvement of cytokines in their proinflammatory effects [47].

Furthermore, aiming to confirm their hypothesis of the involvement of macrophage signaling in the proinflammatory effect of lecithin, Alencar et al. [48] injected *V. macrocarpa* seed lectin into a primary culture of rat macrophages. The culture supernatant was collected and injected into rats’ peritoneal cavities, increasing neutrophil migration proportionally to the lectin concentration. They therefore concluded that *V. macrocarpa* seed lectin can induce cultured macrophages to release a neutrophil chemotactic mediator. These findings highlight the *V. macrocarpa* seed lectin as an important tool for studying pathological conditions associated with excess leukocytes at inflammatory sites causing tissue damage [48]. This also demonstrates an important divergence between the anti-inflammatory effects promoted by the *V. macrocarpa* leaf extract and the proinflammatory effects induced by the seed lectin [24,47,48].

Still investigating the proinflammatory property of lectin, this time obtained from *V. guianensis* seeds, Marques et al. [49] administered it to the rat’s paws, observing the formation of edema in a time- and dose-dependent manner. The researchers suggest that *V. guianensis* lectin binds to specific glycans on targets, triggering inflammatory responses. The COX inhibitor indomethacin and interleukin-1β inhibitor thalidomide partially blocked the edematogenic effect, while the NOS inhibitor L-NAME did not. While pointing to the involvement of prostaglandins and interleukins in lectin-induced edema, this finding excludes the nitric oxide pathway, contrasting with its vasorelaxation mechanism previously discussed [38,49].

#### 4.2.6. Central Nervous System

Notably, lectins are the most studied molecules in glycobiology, and plant lectins can serve as important tools for studying the role of carbohydrate–protein interactions in cellular function modulation. In the central nervous system (CNS) context, it was recently demonstrated that the *V. macrocarpa* seed lectin can alter neural function, eliciting depressive effects and activating neuroinflammatory markers [50]. The study highlights that although it has a carbohydrate affinity, like galactose-1 (gal-1), the lectin can exert neurotoxic effects in the hippocampus of mice, in contrast to the neuroprotective action already reported for gal-1. These findings suggest a possible dual role of galactose-binding lectins in the modulation of CNS function [50]. Lectins can be utilized in both basic and applied research, offering new alternatives for understanding neurological diseases, such as mental disorders, neurodegenerative diseases, and neuro-oncological conditions, as well as driving the search for new therapies for the diagnosis and treatment of these conditions [50,52].

#### 4.2.7. Nociception

Leite et al. [51] studied the effects of *V. macrocarpa* lectin on nociception using menthol- or capsaicin-induced orofacial pain models in zebrafish, the only identified study of this activity for the genus. However, *V. macrocarpa* seed lectin pretreatment failed to demonstrate orofacial antinociception [51].

#### 4.2.8. Wound-Healing

Research on the wound-healing properties of *V. guianensis* has demonstrated its potential to modulate the healing process by affecting the inflammatory response’s intensity and promoting collagen synthesis pathways. This capability indicates that compounds found in the plant may influence tissue regeneration and decrease inflammation related to the healing process [16]. This effect would not be linked only to the pro/anti-inflammatory effect, but also to the antioxidant and antimicrobial activities of the plants. The correlation between these aspects suggests that the modulation of the inflammatory response by *V. guianensis* during the healing process may be partly mediated by the interaction of lectin with glycans on lesioned tissues. This interaction influences the production of essential inflammatory mediators for the initial response to injury that regulates the synthesis of structural components such as collagen, which is crucial to adequate scar tissue formation [16,49]. These hypotheses should be tested in the future, as they may contribute to developing new therapeutic agents and a better understanding of scar modulation strategies.

## 5. Final Considerations

This review is the first to highlight the potential of the *Vatairea* genus, which is utilized in traditional Amazonian medicine and systematizing knowledge about its ethnobotany, traditional knowledge, and uses, but also concerning the scientific evidence regarding its phytochemical composition, pharmacological properties, and safety of its use.

Beginning with its traditional applications, which emphasize its role in treating various skin conditions, it can address numerous pathological states, irritations, wound care, or superficial infections. Interestingly, it has been found that some plant components, such as the lectins present in the seeds, can irritate and stimulate inflammatory processes in peripheral contexts and the central nervous system. However, this stimulating activity of the inflammatory response appears to contribute positively to the evolution of the healing process [13,14,16].

The ability to inhibit the growth of various Gram-positive and Gram-negative bacteria and fungi is an interesting property for treating wounds and skin infections. This effect is likely associated with the antioxidant and anti-inflammatory properties of the phenolic compounds present in the extracts, which could contribute synergistically to the treatment’s overall benefit. This multi-target pattern of drug action has been frequently explored in the search for more effective and safe therapeutic agents and appears to be the case for the *Vatairea* species studied [16,20,22].

In the context of diabetes, for example, the incidence of wounds and skin diseases is critical, given the difficulty in healing associated with the risk of infections that often lead to amputations, and even death. *V. macrocarpa*, in addition to its antimicrobial and anti-inflammatory effects and antioxidant potential, also showed benefits in diabetes models, reducing insulin resistance and glycemia. These effects can be used in isolation and synergistically [26].

Considering also the low toxicity of the extracts of *V. macrocarpa* and *V. guianensis*, it is important to advance in the pharmacological research of the *Vatairea* species, to elucidate their constitutions and properties in the various associated contexts and favor the development of safe and effective therapeutic agents.

## Figures and Tables

**Table 1:** Phytochemical aspects of *Vatairea* genus.

Species	Collection Sites	Plant Parts	Type of Extraction	Phytoconstituents Identified	Reference
*V. guianensis*	Unspecified	Heartwood	Hot extraction Solvent: benzene	Chrysophanic acid-9-anthrone, physcion-9-anthrone and physcion-10-anthrone	[29]
	Ilha do Marapatá, Manaus—Brazil	Stem bark	Soxhlet extraction. Solvent: ethanol	Chrysophanol, physcion, emodin, and triterpenes	[14]
	Parque Ecológico de Porto Velho, Rondônia—Brazil	Fruits	Essential oil hydrodistillation with ethanol	Aldehydes (hexanal, (2Z)-heptenal, (2E,4E)-decadienal, undecenal, dodecanal) and carboxylic acids (docosahexaenoic acid, hexadecanoic acid, and stearic acid)	[18]
	Belém, Pará—Brazil	Leaves	Maceration extraction; Solvent: ethanol	Chrysophanol and physcion	[19]
			Maceration extraction; Solvent: ethanol	5,3′-dihydroxy-4′-methoxy-2″,2″-dimethylpyrano-(5″,6″:8,7)-isoflavone; 5,7-dihydroxy-3′,4′-methylenedioxy-8-prenyl-isoflavone; 5,3′-dihydroxy-4′-methoxy-7-O-β-glucopyranoside-8-prenyl-isoflavone; and derrone	
	Belém, Pará—Brazil	Sapwood	Maceration extraction; Solvent: ethanol	Crysophanol, physcion, formononetin, bolusantol D, betulinic acid, sitosterol, and stigmasterol	[30]
	Belém, Pará—Brazil	Leaves	Maceration extraction; Solvent: ethanol	5,7,3′-trihydroxy-4′-methoxy-8-prenyl-isoflavone; upiwighteone; and 5,7,4′-trihydroxy-3′-methoxy-8-prenyl isoflavone	[22]
*V. heteroptera*	Linhares Forest Reserve, Rio Doce, Espírito Santo—Brazil	Trunk wood	Maceration extraction; Solvent: benzene	Chrysophanol, sitosterol, stigmasterol, emodin, (2S)-7-hydroxiflavone, and formononetin	[31]
*V. macrocarpa*	Campo Grande, Mato Grosso—Brazil	Leaves	Maceration extraction; Solvent: ethanol	Catechin, epicatechin, kaempferol-3-O-α-l-rhamnopyranoside, tannins	[24]

**Table 2:** Pharmacological properties of Vatairea genus.

Experimental Model	Specie	Extract (Part)	Dose/Concentration (via)	Key Outcomes	Ref.
**Toxicity Studies**					
In vitro toxicity					
Leukocyte viability (mice)	*V. macrocarpa*	Ethanolic (leaves)	3–90 μg/mL	No cytotoxicity (MTT).	[24]
Lymphocyte culture (human)	*V. macrocarpa*	Lectin (seed)	0.5–45 µM	Trypan blue assay—concentration-dependent cytotoxicity (≥1 µM)	[43]
			0.5–8 µM	Comet assay: 8 µM—increases DNA damage 0.5–2 µM—decreases doxorubicin-induced DNA damage	
In vivo toxicity					
Acute (male mice)	*V. guianensis*	Hydroethanolic (seed)	2000 and 5000 mg/kg (oral)	No signals of toxicity, or death. LD_50_ > 5000 mg/kg	[16]
	*V. macrocarpa*	Methanolic (heartwood)	100–5000 mg/kg (oral)	No behavioral changes, or death. LD_50_ > 5000 mg/kg	[44]
		Ethanolic (stembark)	250–5000 mg/kg (oral)	No signals of toxicity, or death. LD_50_ > 5000 mg/kg	[26]
Subchronic 30 days (male rats)	*V. macrocarpa*	Methanolic (heartwood)	20–500 mg/kg (oral)	No behavioral, anatomical, or histological changes, either death ↑ Segmented neutrophils (500 mg/kg). ↑ Alkaline phosphatase ↑ Plasma protein ↓ γ-glutamyl transferase (100 mg/kg). ↓ Triacylglyceride	[44]
**Communicable diseases**					
In vitro antibacterial test					
*Enterococcus faecalis*	*V. guianensis*	Hydroalcoholic (seed) Hexane (seed) Chloroform (seed) Methanolic (seed)	0.4–100 μg/mL	MIC 12.5 μg/mL; MBC 25 μg/mL MIC 12.5 μg/mL; MBC 25 μg/mL MIC 3.12 μg/mL; MBC 12.5 μg/mL MIC 12.5 μg/mL; MBC 50 μg/mL	[45]
*Escherichia coli*	*V. macrocarpa*	Lectin (seed)	1.0–1024 μg/mL	No antibiotic activity (MIC ≥ 1024 μg/mL) Decrease norfloxacin antibiotic activity	[42]
*Klebsiella oxytoca*	*V. macrocarpa*	Lectin (seed)	31.25–250 μg/mL	No antibiotic activity (MIC > 250 μg/mL)	[40]
*Pseudomonas aeruginosa*	*V. macrocarpa*	Lectin (seed)	31.25–250 μg/mL	Weakly inhibition of planktonic growth (250 μg/mL).	[40]
	*V. guianensis*	Hydroalcoholic (seed) Hexane (seed) Chloroform (seed) Methanolic (seed)	0.4–100 μg/mL	MIC 25 μg/mL; MBC 100 μg/mL MIC 25 μg/mL; MBC 100 μg/mL MIC 25 μg/mL; MBC 100 μg/mL MIC 25 μg/mL; MBC 50 μg/mL	[45]
*Salmonella* sp.	*V. guianensis*	Hydroalcoholic (seed) Hexane (seed) Chloroform (seed) Methanolic (seed)	0.4–100 μg/mL	No activity No activity MIC 50 μg/mL; MBC 100 μg/mL MIC 50 μg/mL; MBC 100 μg/mL	[45]
*Staphylococcus aureus*	*V. guianensis*	Aqueous (leaves)	2.275 mg/mL (30 μL/hole)	Antibacterial activity at 44.4% of ciprofloxacin (agar diffusion test).	[17]
		Hydroalcoholic (seed) Hexane (seed) Chloroform (seed) Methanolic (seed)	0.4–100 μg/mL	MIC 3.12 μg/mL; MBC 6.25 μg/mL MIC 6.25 μg/mL; MBC 12.5 μg/mL MIC 3.12 μg/mL; MBC 12.5 μg/mL MIC 6.25 μg/mL; MBC 12.5 μg/mL	[45]
	*V. macrocarpa*	Lectin (seed)	1.0–1024 μg/mL	No antibiotic activity (MIC ≥ 1024 μg/mL) Increase in norfloxacin, penicillin, and gentamicin antibiotic activity	[42]
	*V. macrocarpa*	Lectin (seed)	31.25–250 μg/mL	Complete inhibition of planktonic growth (250 μg/mL) Inhibition of biomass formation in biofilms Decrease in the number of viable cells in the biofilm	[40]
*S. epidermidis*	*V. macrocarpa*	Lectin (seed)	31.25–250 μg/mL	Complete inhibition of planktonic growth (250 μg/mL) Influence in biofilm formation Decrease in the number of viable cells in the biofilm	[40]
*Streptococcus sanguis*	*V. macrocarpa*	Lectin (seed)	100 μg/mL	Inhibition of bacterial adhesion to the acquired pellicle on tooth enamel	[41]
In vivo antibacterial test					
*Mycobacterium bovis*	*V. macrocarpa*	Ethanolic (leaves)	30–300 mg/kg	Antimycobacterial activity	[24]
In vitro antifungal test					
*Candida albicans*	*V. macrocarpa*	Ethyl acetate (root bark) Vatacarpan (root bark)	-	MIC 0.98 µg/mL MIC 0.98 µg/mL	[20]
		Lectin (seed)	31.25–250 µg/mL	Weakly inhibition of planktonic growth (250 µg/mL)	[40]
	*V. guianensis*	Ethanolic extract (leaves) Hexanic fraction Ethyl acetate fraction Methanol/H_2_O fraction	0.125–1024 µg/mL	MIC 128 µg/mL; MFC 512 µg/mL No activity MIC 16 µg/mL; MFC 32 µg/mL. No activity	[22]
		5,7,3′-trihydroxy-4′-methoxy-8-prenylisoflavone	0.125–256 µg/mL	No activity	
*C. dubliniensis*	*V. guianensis*	Ethanolic extract (leaves) Hexanic fraction Ethyl acetate fraction Methanol/H_2_O fraction	0.125–1024 µg/mL	MIC 32 µg/mL MIC 64 µg/mL MIC 8 µg/mL; MFC 16 µg/mL No activity	[22]
		5,7,3′-trihydroxy-4′-methoxy-8-prenylisoflavone	0.125–256 µg/mL	MIC 8 µg/mL	
*C. krusei*	*V. guianensis*	Ethanolic extract (leaves) Hexanic fraction Ethyl acetate fraction Methanol/H_2_O fraction	0.125–1024 µg/mL	MIC 128 µg/mL MIC 512 µg/mL; MFC 512 µg/mL MIC 8 µg/mL; MFC 32 µg/mL No activity	[22]
		5,7,3′-trihydroxy-4′-methoxy-8-prenylisoflavone	0.125–256 µg/mL	No activity	
*C. parapsilosis*	*V. macrocarpa*	Ethyl acetate	-	MIC 0.98 µg/mL	[20]
	*V. guianensis*	Ethanolic extract (leaves) Hexanic fraction Ethyl acetate fraction Methanol/H_2_O fraction		No activity MIC 64 µg/mL MIC 8 µg/mL; MFC 32 µg/mL No activity	[22]
		5,7,3′-trihydroxy-4′-methoxy-8-prenylisoflavone	0.125–256 µg/mL	MIC 32 µg/mL	
In vitro antiprotozoal test					
*Leishmania amazonensis*	*V. macrocarpa*	Ethyl acetate (root bark)	-	Antileishmanial activity (IC_50_ 71.47 µg/mL)	[20]
**Non-communicable diseases**					
Endocrine system					
Type 2 diabetes (streptozotocin; male rats)	*V. macrocarpa*	Ethanolic (stembark)	250 and 500 mg/kg (oral, 22 days)	Reductions observed include postprandial glycemia, food and fluid intake, urinary volume, and the excretion of glucose and urea in urine Improvement in weight gain Reduction in HOMA-R index	[26]
			500 mg/kg (oral, 21 days)	Increase insulin receptor and AKT phosphorylation in the liver, extensor digitorum longus muscles, and retroperitoneal white adipose tissue	[27]
Cardiovascular and renal systems					
Ex vivo aortic contraction (rat)	*V. guianensis*	Lectin (seed)	1–100 μg/mL	Concentration-dependent relaxation of phenylephrine-induced aortic contraction This effect appears to involve the release of nitric oxide (NO) by the vascular endothelium Galactose abolishes the lectin’s vasorelaxant effect	[38]
In situ kidney perfusion (rat)	*V. macrocarpa*	Lectin (seed)	10 μg/mL	Increase perfusion pressure, renal vascular resistance, urinary flow, and glomerular filtration rate Galactose abolishes the lectin’s kidney effect Moderate protein buildup in tubules and urinary spaces Renal tubules with eosinophilic casts	[46]
Angiogenic activity Embryo chorioallantoic membrane (chicken)	*V. macrocarpa*	Lectin (seed)	0.5–8 μM	↑ Vascularization and number of blood vessels (angiogenesis) ↑ Length, size, number of complexes, and blood vessel junctions ↑ Inflammatory cells and fibroblasts ↑ Thickening of CAM ↑ VEGF and TNF-α expression Lactose reduced the lectin’s effects	[43]
Immune system					
In vitro inflammation					
Neutrophil phagocytic activity	*V. macrocarpa*	Ethanolic (leaves)	3–30 μg/mL	Reduction in neutrophil phagocytic activity	[24]
In vivo inflammation					
Carrageenan-induced pleurisy, BCG-induced pleurisy, CFA-induced paw edema	*V. macrocarpa*	Ethanolic (leaves)	10–300 mg/kg (oral)	Dose-dependent reduction in leukocyte migration and protein concentration in pleural exudate CFA-induced paw edema: no effect in hyperalgesia, reduction in paw edema, and cold sensitivity	[24]
Neutrophil migration (female rat)	*V. macrocarpa*	Lectin (seed)	9.6 × 10^−7^, 1.9 × 10^−6^, or 3.8 × 10^−6^ M (1 mL, intraperitoneal)	Neutrophil and mononuclear cell migration to the peritoneal cavity is induced in a dose-dependent manner through macrophage-mediated mechanisms (cytokine release) Galactose abolishes the lectin’s proinflammatory effect, suggesting it acts via its carbohydrate-binding site.	[47]
	*V. macrocarpa*	Lectin (seed)	4.8 × 10^−7^, 9.6 × 10^−7^, or 1.9 × 10^−6^ mol	Lectin induces cultured macrophages to release a neutrophil chemotactic mediator	[48]
Paw edema (rat)	*V. guianensis*	Lectin (seed)	0.01, 0.1, and 1 mg/kg	Time- and dose-dependent paw edema, with polymorphonuclear infiltrate Indomethacin (COX blocker) partially inhibits this effect, but L-NAME (NOS inhibitor) does not	[49]
Central nervous system					
Neuroinflammation (male mice)	*V. macrocarpa*	Lectin (seed)	0.3–3 μg/site (intracerebroventricular)	Depressive-like effect (forced swimming test) Proinflammatory effect in the hippocampus: (↑ COX-2, GFAP, and S100B)	[50]
Nociception					
Orofacial nociception (zebrafish)	*V. macrocarpa*	Lectin (seed)	0.025, 0.05 or 0.1 mg/mL (20 μL; intraperitoneal)	No pain relief effect	[51]
Wound-healing					
Dorsal wound (rat)	*V. guianensis*	Hydroethanolic (seed)	100–500 mg/kg (topical)	Improve wound contraction from the third day of treatment (100 mg/kg) Inflammatory response reduction Stimulation of collagen synthesis	[16]

## Data Availability

No new data were created or analyzed in this study. Data sharing is not applicable to this article.
